# Formaldehyde Exposure and Lower Respiratory Infections in Infants: Findings from the PARIS Cohort Study

**DOI:** 10.1289/ehp.1003222

**Published:** 2011-08-02

**Authors:** Célina Roda, Isabelle Kousignian, Chantal Guihenneuc-Jouyaux, Claire Dassonville, Ioannis Nicolis, Jocelyne Just, Isabelle Momas

**Affiliations:** 1 Université Paris Descartes, Département Santé Publique et Biostatistiques – EA 4064, Laboratoire Santé Publique et Environnement, Paris, France; 2 Centre de l’Asthme et des Allergies, Groupe Hospitalier Trousseau – La Roche Guyon, Assistance Publique – Hôpitaux de Paris, Paris, France; 3 Mairie de Paris, Direction de l’Action Sociale de l’Enfance et de la Santé, Cellule Cohorte, Paris, France

**Keywords:** birth cohort, epidemiology, exposure, formaldehyde, lower respiratory infection, predictive model

## Abstract

Background: Certain chemical pollutants can exacerbate lower respiratory tract infections (LRIs), a common childhood ailment. Although formaldehyde (FA) is one of the most common air pollutants found in indoor environments, its impact on infant health is uncertain.

Objective: Our aim was to determine the impact of FA exposure on the LRI incidence during the first year of life of infants from the Pollution and Asthma Risk: an Infant Study (PARIS) birth cohort.

Methods: FA was measured in a random sample of 196 infants’ dwellings, and exposure to this pollutant was estimated for 2,940 infants using predictive models based on measurements and data about potential determinants of FA levels. Health data were collected from parents by regular self-administered questionnaires. We used multivariate logistic regressions to estimate associations between FA exposure and the occurrence of LRI and wheezy LRI (wLRI), adjusting for potential confounders/risk factors.

Results: During the first year of life, 45.8% of infants had at least one LRI, and LRI occurred simultaneously with wheezing in 48.7% of cases. The FA predictive models correctly classified 70% of dwellings as having high or low exposure, and we estimated that 43.3% of infants were exposed throughout the first year to levels of FA > 19.5 µg/m^3^. FA exposure was significantly associated with LRI and wLRI before and after adjustment for known LRI risk factors/confounders. For an interquartile increase in FA levels (12.4 μg/m^3^), we estimated a 32% [95% confidence interval (CI): 11, 55] and 41% (95% CI: 14, 74) increase in the incidence of LRI and wLRI, respectively.

Conclusion: The findings of this study suggest that infants exposed to FA at an early age have an increased incidence of LRI.

Lower respiratory tract infections (LRIs) are common in infants and are induced mainly by viral exposure. LRIs can cause wheezing and may be responsible for long-term effects on airway function (Gern and Busse 2000; Martinez et al. 1995). Certain LRI risk factors have been identified, such as day-care attendance (Koopman et al. 2001), presence of older siblings (Koopman et al. 2001), and parental history of asthma (Bosken et al. 2000; Goetghebuer et al. 2004). Besides these well-known causes, environmental exposure during the first year of life has been suggested as having an important impact on infant respiratory health, because of the vulnerability of young children due to their growth, the immaturity of their organs, the development of their immune system, their behavior (hand-to-mouth contact), and exposure conditions (e.g., time spent indoors). Potential links between child exposure and chemical pollutants such as environmental tobacco smoke (ETS), styrene, benzene, nitrogen dioxide (NO_2_), ozone (O_3_), and the risk of respiratory infections have been described (Chauhan et al. 2003; Diez et al. 2000; Strachan and Cook 1997; Tepper et al. 2005). Furthermore, a recent prospective cohort showed that high exposure to NO_2_, assessed by personal measurement before infection, is associated with increased severity of lower respiratory symptoms (Chauhan et al. 2003).

These findings led us to question the association between other indoor chemical pollutants and LRI. Formaldehyde (FA) is omnipresent in indoor environments where infants spend most of their time. This aldehyde is known to be an irritant to the eye as well as to the upper and lower respiratory airways (Wolkoff and Nielsen 2010). The effect of FA exposure on infants’ health is unclear (Garrett et al. 1999; Mendell 2007; Raaschou-Nielsen et al. 2009; Rumchev et al. 2002; Tavernier et al. 2006), because little research has been conducted to date. A recent meta-analysis showed a significant positive association between FA exposure and asthma in children with a pooled odds ratio (OR) of 1.03 [95% confidence interval (CI): 1.02, 1.04] for an increase of 10 μg/m^3^ (McGwin et al. 2010). A Danish clinical birth cohort, the Copenhagen Prospective Study on Asthma in Childhood (COPSAC), which enrolled newborns of mothers with asthma, found no relation between FA exposure and the incidence of wheezing (Raaschou-Nielsen et al. 2009). Conversely, other authors have reported evidence of an airway inflammatory response to FA exposure based on exhaled nitric oxide levels in healthy children between 6 and 13 years of age (Franklin et al. 2000).

The aim of this study was thus to assess, in the Pollution and Asthma Risk: an Infant Study (PARIS) birth cohort, the influence of FA exposure on LRI, especially wheezy LRI (wLRI), during the first year of life.

## Materials and Methods

*Study design.* The prospective follow-up of the PARIS birth cohort was based on parent self-administered questionnaires completed when infants were 1, 3, 6, 9, and 12 months of age and at each birthday until age 7 years, and on two health examinations at 18 months and 7 years of age. Here we describe data related to the first year of life.

*Study population.* The PARIS cohort enrolled 4,177 healthy newborns recruited in five maternity hospitals in Paris, France, from 2003 to 2006 according to medical and sociodemographic criteria described elsewhere (Clarisse et al. 2007). Eligibility criteria included singleton full-term newborns with a birth weight > 2,500 g and an uncomplicated birth and neonatal period. Parents had to reside in the Paris area or its close suburbs, and the mothers had to speak French. Of the 4,177 newborns initially enrolled in the study, 337 did not return any mailed questionnaires, resulting in a final cohort of 3,840 children. This study was approved by the National Ethics Committee (permissions 031153 and 051289), and parents of participating infants gave their written informed consent.

*Data collection.* Health data. At birth, an interview with the mother was conducted to collect data about the history of allergic conditions (asthma, atopic dermatitis, and allergic rhinitis) in both parents. Sex of the child, parity, anthropometric parameters of the child, and maternal history of pregnancy and delivery were also registered from medical records of the newborn and mother.

Parents regularly documented health outcomes in mailed questionnaires. Most questions were derived from previously validated questionnaires, such as the Asthma Multicentre Infants Cohort Study (AMICS) (Sunyer et al. 2004) and the French version of the International Study on Allergies and Asthma in Childhood (ISAAC) (Charpin et al. 1998). Parents were questioned on the occurrence of infectious diseases (upper respiratory infections, i.e., colds and ear infections; and LRI, i.e., bronchitis, bronchiolitis), symptoms evocating eczema, episodes of wheezing and whistling in the chest, and the frequency of these episodes (any wheeze, from 1 to 3, from 4 to 12, or > 12 wheezing episodes) at different periods: from birth to 1 month, from 1 to 3 months, from 3 to 6 months, from 6 to 9 months, and from 9 to 12 months. In addition, at 1 year of age, responses concerning obstructive symptoms such as shortness of breath, dyspnea responsible for sleep disturbance, and dry cough at night apart from a cough associated with a cold or chest infection were recorded.

Environmental and lifestyle data. One month after birth, a standardized phone interview was conducted with parents by a trained interviewer to determine home characteristics (construction date, number of occupants, home surface area, heating and cooking appliances, presence of mechanical ventilation and double glazing, wall and floor coverings, and signs of dampness) and family living conditions [duration of breast-feeding, information on day-care attendance, keeping of pets, aeration, smoking, use of air fresheners, do-it-yourself (DIY) activities such as home improvement, decorating, crafts, lifestyle and recreation, scale modeling]. The interview also included data on potential continuous sources of FA levels (presence and age of wood-pressed product for flooring or varnished parquet floor, particle board furniture, wall coating). When the infant was 3, 6, 9, and 12 months of age, an environmental questionnaire was sent to parents to record changes in home and family life. If families moved, a new questionnaire was administered to characterize the new dwelling.

Measurements of FA levels. An environmental investigation was conducted on a random sample of 196 infants’ homes from the PARIS birth cohort to assess exposure to indoor pollutants and to measure FA. Ten infants moved during the follow-up year, resulting in 206 dwellings being investigated.

Aldehyde air-sampling measurements were performed in the selected infants’ homes four times during the first year of life at 1, 6, 9, and 12 months. FA was collected using a passive sampler (Radiello; Fondazione Salvatore Maugeri-IRCCS, Padua, Italy) placed in the bedroom of the infant for 7 days. The passive sampler was a stainless steel net cartridge filled with 2,4-dinitrophenylhydrazine (2,4-DNPH)–coated Florisil. Aldehydes react with 2,4-DNPH to give the corresponding 2,4-dinitrophenylhydrazones. After 7 days of exposure, each cartridge was sent to the laboratory and kept at 4°C until analysis. The analyses were performed by high-performance liquid chromatography and ultraviolet detection at wavelength of 365 nm (Perkin-Elmer series 200UV/visible detector; PerkinElmer Life and Analytical Sciences, Shelton, CT, USA). Detection and quantification limits were 0.8 µg/m^3^ and 2.4 µg/m^3^, respectively, and the quality insurance measurements has been described previously (Dassonville et al. 2009).

*Data analysis.* Statistical analyses were performed with STATA statistical software (release 9.2; Stata Corporation, College Station, TX, USA). A *p*-value < 0.05 was considered to be statistically significant. Sensitivity analysis was implemented using WinBUGS software (Lunn et al. 2000).

Modeling of domestic FA exposure. FA exposure was determined building predictive models using data from the environmental investigation (FA measurements and questionnaires) through two approaches: *a*) a linear regression model to estimate continuous FA levels and *b*) a logistic regression model to predict categorical FA levels.

For each home visited, we calculated an annual mean FA level defined as the average of the seasonal levels to take into account FA seasonal variations. We used median and tertile levels to define two or three categorical levels of FA. Potential predictive factors were continuous FA sources (presence and age of wood-pressed product for flooring or varnished parquet floor, particle board furniture, wall coating in the infant’s bedroom), discontinuous FA sources (smoking, frequency of using air fresheners and cleaning products, DIY activities), home characteristics (construction date, number of occupants, home surface area) as a surrogate for other FA sources, and parameters that might influence indoor concentration of pollutant (frequency of aeration and length of window opening, presence of a mechanical ventilation system, presence of double glazing).

Relations between each potential factor and FA levels or classes were examined, and variables with a univariate *p*-value of ≤ 0.20, in addition to factors identified in earlier studies as potential confounders, were entered in multivariate regression models.

To predict continuous FA levels using the linear regression approach, observed FA levels were log-transformed because of a log-normal distribution. We measured colinearity among variables in the model by the variance inflation factor. We assessed the probability of being in the “high” FA class defined by a level above the median or the upper FA tertile using a logistic regression model and a multinomial logistic regression model. We used Akaike information criterion values to select the parsimonious model. And we assessed goodness of fit using the Hosmer–Lemeshow test.

To evaluate model discrimination performance, we used diagnostic criteria such as sensitivity, specificity, and the area under the receiver operating characteristics curves (AUC). To express model performance of linear regression as in the logistic regression, we classified predicted levels with reference to the median of observed FA levels, and we compared predicted and observed classes.

The predictive models were then applied to dwellings of all cohort members, and exposure to FA was determined for all children, taking into account any changes in residence.

Study of the associations between LRI/wLRI and FA exposure. The considered outcomes were the occurrence of LRI and wLRI during the first 12 months. Potential risk factors included sex, older siblings (fewer or more than two older siblings), and parental asthma history (none, one, or both parents). Socioeconomic status (SES), defined as the highest level occupation of the two parents, was categorized into high level (high-level white-collar workers), medium level (intermediate white-collar workers, craftsmen, and shopkeepers), and low level (low-level white-collar workers, blue-collar workers, and unemployed). Prenatal tobacco smoke exposure was characterized by active or passive smoking at home or at work during pregnancy, and postnatal tobacco smoke exposure was defined as the presence of a regular smoker in the home (smoking every day). A composite variable representing the number of signs of dampness (damp stains, mold stains, water damage, and/or mold odor) was included. Length of exclusive breast-feeding (less or more than 3 months), day care center attendance (yes or no), and presence of pets at home (yes or no) were also considered.

Association between LRI and FA exposure (expressed either as continuous or categorical variable) was first analyzed by unconditional logistic regression after 1 year of follow-up. Three mutually exclusive patterns of LRI were then considered in a multinomial logistic regression: “never LRI” (no LRI during the first year of life), “non-wLRI” (LRI without wheezing), and “wLRI” (LRI and wheezing recorded simultaneously). The model was fitted using “never LRI” as the reference stratum, and all variables with a *p*-value of ≤ 20% in univariate analyses and potential confounders described in the literature were included in the multivariate model. Results were expressed as adjusted ORs with their 95% CIs.

Sensitivity analysis: multiple imputation. Because a single imputation strategy based upon regression predictions (as above mentioned) could underestimate the standard errors of estimates, we also used multiple imputation techniques for missing data, previously described by Rubin (1987) and Little and Rubin (2002). These techniques have a double advantage in that they restore variability in the missing data and take into account the uncertainty due to simulating missing data.

As suggested by Carpenter and Kenward (2005), multiple imputation including health outcome was implemented in a Bayesian context. This approach is less conservative than classical imputation techniques and is referred to in the literature as “fully Bayesian model.” It extends multiple imputation by jointly simulating the distributions of variables with missing data as well as unknown parameters in a regression equation, as previously described by Carrigan et al. (2007). We assumed a missing completely at random mechanism for unmeasured values of FA, because families where measurements were carried out were selected at random. As mentioned by Allison (2009), when data are missing by design, this is the best situation in which the assumption is likely to be satisfied. Furthermore, we performed univariate and multivariate analyses, which give results very consistent with missing at random assumption. First, we assessed association between each predictive factor of FA levels and missing status (denoted R) using chi-square tests of independence or Student *t*-tests applying Bonferroni correction, and no significant association was found. Second, a global test was implemented, that is, a multivariate logistic regression of R on all predictive factors was conducted and compared with a model without these factors; there was not any significant difference between these models.

The sensitivity analysis consisted of multiple imputation and health model jointly fitted using Markov chain Monte Carlo (MCMC) methods (Gilks et al. 1996). The algorithm was run for 10,000 iterations with 1,000 iterations discarded for burn-in. Because MCMC is an iterative procedure, it is essential that MCMC convergence is achieved. The convergence of the algorithm was assessed by convergence diagnostics. Inspection of posterior plots of the iteration versus the generated values as well as correlation plots indicated that convergence was not an issue. Results were reported as posterior mean of OR with 95% credibility interval (95% Cr).

## Results

Results are given for 2,940 infants for whom FA exposure estimates and information about LRI were available at 1 year of age.

*Characteristics of the study population.* Table 1 shows characteristics of the study population at birth. Newborns were often the first baby of the family (55.9%) and came from high-SES families (65.5%). Among the infants, 576 (19.6%) had a parental history of asthma. At least one parent was a regular smoker (every day) in 20.1% of households, and 10.4% of babies were exposed *in utero* to ETS. Half of the infants were exclusively breast-fed (52.6%) at 1 month of age, and 9.1% were breast-fed for > 3 months. Around half of infants (43.2%) attended a child day-care center, and 56.1% of them entered nursery before 6 months of age. Furry pets were present in 18.8% of households.

**Table 1:** Characteristics of infants from the PARIS cohort study at birth (*n* = 2,940).

Characteristic	No. (%)
Male sex	1,510 (51.4)
Siblings	
None	1,644 (55.9)
1 older sibling	1,008 (34.3)
≥ 2 older siblings	288 (9.8)
Parental social economic status*a*	
High level	1,925 (65.5)
Median level	789 (26.8)
Low level	226 (7.7)
Parental asthma history	
No	2,354 (80.1)
One parent	546 (18.6)
Two parents	30 (1.0)
Unknown	10 (0.3)
**a**High level: high-level white-collar workers; median level: intermediate white-collar workers, craftsmen, and shopkeepers; low level: low-level white-collar workers, blue-collar workers, and unemployed.	

Table 2 gives characteristics of dwellings and living conditions that are the potential determinants of FA levels. Overall, the main characteristics of dwellings in our environmental investigation did not differ from the characteristics of other dwellings in the cohort. Most infants lived in apartments (92.3%) with a mean (± SD) area of 71.4 ± 25.9 m^2^, and nearly one-third of buildings were built after 1975 (28.2%). Around half (48.8%) and two-thirds (66.6%) of babies had wood-pressed products for flooring or varnished parquet floor and particle board furniture in their bedroom, respectively. Signs of dampness were noted in 29.7% of homes. Around 15% of parents declared using air fresheners and cleaning products at least once a week, and 12.2% reported DIY activities in the home. During the first year of life, 10.3% of newborns from the PARIS cohort changed residences, and 303 additional dwellings were frequented, so 3,243 dwellings were attended at least once by the newborns.

**Table 2:** Potential determinants of FA levels in the PARIS cohort study at birth (*n* = 2,940) and in the environmental investigation (*n* = 196).

Potential determinants	Overall dwellings *n* (%)	Environmental investigation *n* (%)
Type of home				
Apartment		2,713 (92.3)		182 (92.9)
House		227 (7.7)		14 (7.1)
Construction date				
Before 1975		2,112 (71.8)		142 (72.5)
1976–1990		358 (12.2)		22 (11.2)
After 1990		470 (16.0)		32 (16.3)
Housing area [m^2^ (mean ± SD)]		71.4 ± 25.9		71.3 ± 25.2
Wood-pressed products for flooring or varnished parquet floor				
No		1,505 (51.2)		93 (47.4)
Yes		1,435 (48.8)		103 (52.6)
Wall coating (paint or fiber cloth)				
No		583 (19.8)		54 (27.6)
Yes		2,357 (80.2)		142 (72.4)
Particle board furniture				
No		982 (33.4)		57 (29.1)
Yes		1,958 (66.6)		139 (70.9)
Mechanical ventilation				
No		2,219 (75.5)		145 (74.0)
Yes		721 (24.5)		51 (26.0)
Double glazing				
No		1,051 (35.7)		75 (38.3)
Yes		1,889 (64.3)		121 (61.7)
Humidity sign				
No		2,066 (70.3)		114 (58.2)
Yes		874 (29.7)		82 (41.8)
No. of occupants in individual home (mean ± SD)		3.6 ± 0.8		3.5 ± 0.7
Smoking at home		592 (20.1)		46 (23.5)
Frequency of use of air fresheners and cleaning products				
Less than once a week		2,509 (85.3)		157 (80.1)
At least once a week		431 (14.7)		39 (19.9)
DIY activities		359 (12.2)		33 (16.8)
Values are *n* (%) except where noted.				

*FA exposure.* In the environmental investigation, the annual FA level was defined for 174 dwellings, with a median equal to 19.5 µg/m^3^ [interquartile range (IQR): 14.4–26.8].

Table 3 shows ORs of the multivariate logistic regression and coefficients of the multivariate linear regression associated with predictive factors of FA exposure classes and FA levels, respectively. The model included continuous sources of FA (presence and age of wall coating, wood-pressed products for flooring or varnished parquet floor, and particle board furniture) and parameters of aeration and air stuffiness that might influence FA emissions from materials, such as length of window opening, presence of mechanical ventilation and double glazing. Home characteristics, such as construction date, housing area, and number of occupants were also included. The logistic model showed satisfying goodness of fit (Hosmer–Lemeshow test, *p* > 0.05). Examining the agreement between predicted and observed exposure classes, 73.0% of dwellings were correctly classified as high or low FA, with sensitivity and specificity of 72.4% and 73.6%, positive and negative predictive values of 73.3% and 72.7%, respectively, and an AUC of 0.81. Predicting three FA classes defined by tertiles, the amount of agreement between the true and predicted data in the highest tertile was 67.2%, and sensitivity and specificity were 57.4% and 82.1%, respectively. As for logistic regression, linear regression model accurately classified 70% of dwellings.

**Table 3:** Predictive models of FA exposure class and levels in the PARIS cohort study by multivariate logistic and linear regression models.

Multivariate logistic regression model			Multivariate linear regression model		
Predictive factors	OR (95% CI)	*p*	β (95% CI)	*p*
Home characteristics								
Construction date								
1976–1990 (vs. before 1975)		1.26 (0.41, 3.92)		0.686		0.04 (–0.17, 0.24)		0.722
After 1990 (vs. before 1975)		3.61 (1.09, 11.98)		0.036		0.09 (–0.09, 0.27)		0.334
Housing area								
≥ 70 m² (vs. < 70 m²)		2.07 (0.94, 4.58)		0.070		0.10 (–0.03, 0.23)		0.141
No. of occupants								
> 3 (vs. ≤ 3)		2.11 (0.96, 4.64)		0.064		0.12 (–0.02, 0.25)		0.086
Continuous sources								
Wall coating (paint or fiber cloth)								
Yes ≥ 1 year (vs. no)		5.34 (1.84, 15.46)		0.002		0.16 (–0.01, 0.33)		0.053
Yes < 1 year (vs. no)		5.14 (1.76, 15.03)		0.003		0.17 (–0.01, 0.34)		0.052
Wood-pressed products for flooring or varnished parquet floor								
Yes ≥ 1 year (vs. no)		1.98 (0.87, 4.51)		0.103		0.15 (0.01, 0.29)		0.037
Yes < 1 year (vs. no)		3.70 (1.06, 12.86)		0.040		0.23 (0.03, 0.44)		0.023
Particle board furniture								
Yes ≥ 1 year (vs. no)		4.20 (1.16, 15.17)		0.028		0.37 (0.16, 0.58)		0.001
Yes < 1 year (vs. no)		4.34 (1.30, 14.53)		0.017		0.42 (0.23, 0.61)		< 0.001
Aeration/air stuffiness								
Mechanical ventilation								
Yes (vs. no)		1.74 (0.72, 4.21)		0.221		0.08 (–0.06, 0.23)		0.266
Double glazing								
Yes (vs. no)		2.76 (1.22, 6.28)		0.015		0.28 (0.14, 0.42)		< 0.001
Duration of windows opened (> 1 hr)*a*		0.89 (0.81, 0.99)		0.028		–0.02 (–0.03, 0.01)		0.061
β, partial regression coefficient. **a**Adjusted for season of declaration.								

After applying these models to all dwellings of PARIS cohort members, we estimated that infants were exposed to an average (± SD) annual FA level of 19.5 ± 5.4 μg/m^3^. Around 48% and 39% of infants were exposed at least once during their first year of life to FA level above the median and the upper FA tertile; 43.3% and 35.2% were in the two highest exposure classes for 12 months.

*LRI and wLRI occurrence.* Overall, 45.8% of infants had at least one LRI during the first year of life, and 48.7% of these LRI occurred simultaneously with wheezing.

*Relation between FA exposure and LRI and wLRI onset.* Breast-feeding for at least 3 months and presence of furry pets at home were inversely associated with occurrence of LRI (Table 4). Conversely, male sex, parental history of asthma, presence of older siblings, and day-care attendance were positively significantly associated with LRI and wLRI. Prenatal exposure to ETS was positively associated with the risk of infections, whereas postnatal exposure had a weaker estimated impact. After adjustment for these risk factors, an interquartile increase in FA levels (12.4 µg/m^3^) was associated with a 32% (95% CI: 1.11, 1.55; *p* = 0.001) and 41% (95% CI: 1.14, 1.74; *p* = 0.001) increase in risk of LRI and wLRI, respectively. The adjusted ORs related to exposure above the FA median and the upper tertile were 1.20 (95% CI: 1.03, 1.41; *p* = 0.020) and 1.31 (95% CI: 1.10, 1.57; *p* = 0.003) for LRI, and 1.31 (95% CI: 1.07, 1.59; *p* = 0.007) and 1.43 (95% CI: 1.14, 1.79; *p* = 0.002) for wLRI, respectively.

**Table 4:** Association between FA exposure and LRI or wLRI, with adjustment for potential risk factors at 1 year of life in PARIS cohort study.

LRI*a*			wLRI*b*		
Variables	OR (95% CI)	*p*	OR (95% CI)	*p*
Exposure to FA (↑ IQR)		1.32 (1.11, 1.55)		0.001		1.41 (1.14, 1.74)		0.001
Prenatal exposure to ETS (yes vs. no)		1.21 (1.01, 1.45)		0.049		1.32 (1.05, 1.67)		0.017
Postnatal exposure to ETS (yes vs. no)		1.05 (0.85, 1.29)		0.656		1.21 (0.93, 1.56)		0.149
Breast-feeding (≥ 3 months vs. < 3 months)		0.76 (0.63, 0.90)		0.002		0.79 (0.64, 0.99)		0.037
Siblings (≥ 2 vs. < 2)		1.36 (1.05, 1.77)		0.021		1.61 (1.18, 2.21)		0.003
Day-care attendance (yes vs. no)		2.31 (1.98, 2.70)		< 0.001		2.69 (2.21, 3.28)		< 0.001
Furry pets (yes vs. no)		0.81 (0.67, 0.97)		0.025		0.66 (0.51, 0.84)		0.001
Humidity score								
1 sign vs. no		1.04 (0.82, 1.30)		0.760		1.10 (0.83, 1.46)		0.521
≥ 2 signs vs. no		1.15 (0.93, 1.43)		0.203		1.36 (1.05, 1.77)		0.021
Sex (male vs. female)		1.33 (1.14, 1.55)		< 0.001		1.45 (1.19, 1.76)		< 0.001
Parental asthma (yes vs. no)		1.08 (0.89, 1.31)		0.452		1.42 (1.13, 1.79)		0.003
↑, increase. **a**Logistic regression model (outcome: no LRI/LRI) with adjustment also for socioeconomic status (no significantly associated). **b**Multinomial logistic regression model (outcome: no LRI/non-wLRI/wLRI) with adjustment also for socioeconomic status (not significantly associated).								

Furthermore, associations between FA exposure and wLRI were confirmed in sensitivity analysis: After multiple imputation for missing values, adjusted ORs associated with an interquartile increase in FA levels were 1.16 (95% Cr: 1.04, 1.29) and 1.19 (95% Cr: 1.06, 1.32) for LRI and wLRI, respectively.

## Discussion

This study provides, for the first time, data on the association between FA exposure and occurrence of LRI, especially wLRI, in a large birth cohort. The role of long-term FA exposure in homes and/or schools is poorly documented and not yet established in childhood. Some authors have reported a positive association between FA exposure and prevalence/incidence of respiratory symptoms (Garrett et al. 1999; Hulin et al. 2010; Rumchev et al. 2002; Smedje et al. 1997; Venn et al. 2003), whereas others did not find any association (Kim et al. 2007; Mi et al. 2006; Raaschou-Nielsen et al. 2009; Smedje and Norback 2001; Tavernier et al. 2006). This association thus remains uncertain, mainly because of the difficulty in assessing FA exposure.

Most authors interested in the health impact of FA exposure on children have carried out environmental investigations that include indoor and/or outdoor measurements of pollutants. These studies have generally dealt with only a few hundred children because of technical constraints and the significant cost (Khalequzzaman et al. 2007; Tavernier et al. 2006; Venn et al. 2003). Measurements have either been unique (Franklin et al. 2000; Hulin et al. 2010; Khalequzzaman et al. 2007; Tavernier et al. 2006; Venn et al. 2003) or repeated (Delfino et al. 2003; Garrett et al. 1999; Krzyzanowski et al. 1990; Raaschou-Nielsen et al. 2009; Rumchev et al. 2002), which is more representative of chronic exposure. In addition, a strong seasonal effect has been described previously, with FA levels being higher in the hot season than in the cold season (Dassonville et al. 2009; Wolkoff and Nielsen 2010). In large-scale epidemiologic studies, authors have assessed exposure to indoor pollutants using environmental questionnaires and items related to the renovation of the child’s room and presence of FA sources such as particle board (Jaakkola et al. 2004).

As far as we know, no team has modeled FA exposure to classify subjects involved in an epidemiological survey. We have tried to combine both approaches generally used to estimate exposure to FA (measurements and questionnaires data) to build a predictive model of FA levels that could be applied to subjects involved in an epidemiological survey. We are aware that this approach does have limitations. First, the model is based on only a few hundred measurements. However, measurements were repeated and conducted on a random sample of children from the PARIS cohort, with their dwellings being representative of dwellings of all cohort members. In addition to continuous FA levels, the median FA level was used to define two exposure classes based on a threshold that also corresponds to the median FA level in the French nationwide study conducted by the Indoor Air Quality Observatory (Kirchner et al. 2007). The model, which accurately classified > 70% of dwellings, is consistent with the literature, because it includes indoor sources of FA such as particle board furniture, presence of wood-pressed products for flooring, or varnished parquet floor. These sources have been described as being the most important according to emission data (Bruinen de Bruin et al. 2005; Salthammer et al. 2010) and have also been identified as determinants of FA levels measured indoors (Clarisse et al. 2003; Dassonville et al. 2009; Gilbert et al. 2006). As expected, FA levels tended to decrease with aeration and to increase with double glazing, with air stuffiness increasing material emissions (Clarisse et al. 2003; Gilbert et al. 2008). Another factor that could limit the predictive value of the model could be that data were collected by self-administered questionnaires. In large-scale studies, it is not possible to measure air exchange rates. Nevertheless, the duration of window opening reported by parents was inversely associated with FA levels. Items such as building age and mechanical ventilation are likely to be surrogates of other FA sources not identified in our survey.

Respiratory infections are the most common cause of child illness throughout the world. In infancy, viral respiratory infections have been frequently reported with wheezing (Bosken et al. 2000), which has led some authors such as Martinez to distinguish wLRI and non-wLRI and to consider wheeze as a symptom of severity of LRI (Martinez et al. 1995). In our study, health data were self-reported, and it was not possible to detect viruses in samples of nasal secretion in all children. Nevertheless, the reported incidence of LRI (and wLRI) in our cohort, 45.8% (and 22.3%) during the first year of life, is consistent with previous studies (Puig et al. 2008). Furthermore the occurrence of respiratory manifestations was prospectively recorded by repeated questionnaires mailed at frequent intervals, at 1 month of age and then every 3 months until the first birthday, so that the risk of recall bias was limited. The quality of our data is further supported by confirmation of established risk factors for LRI such as sex of child (Taussig et al. 2003); parental history of asthma (Linneberg et al. 2006); presence of older siblings and day-care attendance, which evoke exposure to viral agents (Ball et al. 2000); signs of humidity (Jaakkola et al. 2005); the protective effect of breast-feeding (Duijts et al. 2009); and the impact of ETS exposure. Besides these known risk factors, we found an association between FA exposure and the occurrence of wLRI. These associations were confirmed using a multiple imputation approach.

FA is known to be an irritant of airways. Domestic FA exposure increases respiratory symptom severity among children with wheezing illness (Venn et al. 2003).

FA exposure could particularly affect the airways of babies because of their susceptibility at this young age and the fact that more air is inhaled because of a faster breathing rate. Daily inhalation rates of newborns have been shown to be 2.1–5.1 times higher than those of adults (Brochu et al. 2006). Our results are consistent with the impact of other chemical pollutants (i.e., ETS, NO_2_, O_3_, styrene) on respiratory infections. An increase in risk of serious respiratory infection associated with ETS exposure among children 0–6 years of age has been shown (Li et al. 1999), with the pooled OR being 1.57 (95% CI: 1.28, 1.91). Fusco et al. (2001) found that hospital admissions for respiratory infections in Italian children were significantly associated with NO_2_ levels (4.0% increase per interquartile change; 22.3 µg/m^3^) and O_3_ levels (5.4% increase per interquartile change; 23.9 µg/m^3^). In a German study (Diez et al. 2000), styrene levels > 2 µg/m^3^ (levels 15 times lower than the threshold value in the indoor air in Germany, 30 µg/m^3^) were associated with the risk of respiratory infections in children 6 weeks of age (OR = 2.1; 95% CI: 1.1, 4.5). The authors explain that babies have irritable mucosae; some biological mechanisms may be responsible for the increase of LRI in young infants exposed to these pollutants. Defense mechanisms of the bronchial tree such as mucociliary clearance could be impaired by exposure to these pollutants, thus increasing susceptibility to pathogens such as viruses. Pollutants might also disrupt epithelial cell tight junction complexes and increase virus entry (Ciencewicki and Jaspers 2007). These mechanisms might explain how FA increases the incidence of respiratory infections.

In summary, our data suggest a link between chronic exposure to FA and the occurrence of LRI during the first year of life in healthy Paris newborns from a large epidemiological study. This association persists after adjustment for all potential risk factors. Infections occurring during infancy have been hypothesized to have severe repercussions on respiratory function, because this infection occurs during the phase of lung growth. It is therefore important that children in our study be followed up. It is possible that exposure to FA could be limited by decreasing the use of FA-emitting materials and increasing aeration in dwellings during this sensitive period in the child’s life.
